# ETDRS panretinal photocoagulation combined with intravitreal ranibizumab versus PASCAL panretinal photocoagulation with intravitreal ranibizumab versus intravitreal ranibizumab alone for the treatment of proliferative diabetic retinopathy

**DOI:** 10.5935/0004-2749.20200096

**Published:** 2024-02-11

**Authors:** Rafael de Montier P. Barroso, Katharina Messias, Denny Marcos Garcia, José Augusto Cardillo, Ingrid U. Scott, Andre Messias, Rodrigo Jorge

**Affiliations:** 1 Department of Ophthalmology, Faculdade de Medicina de Ribeirão Preto, Universidade de São Paulo, Ribeirão Preto, SP, Brazil; 2 Departments of Ophthalmology and Public Health Sciences, Penn State College of Medicine, Hershey, PA, USA

**Keywords:** Diabetic retinopathy, Retina, Diabetes, Laser, Vascular endothelial growth factor A, Angiogenesis inhibitors/therapeutic use, Ranibizumab/therapeutic use, Panretinal photocoagulation, Visual acuity, Retinopatia diabetica, Retina, Diabetes, Fator A de crescimento do endotélio vascular, Inibidorres da angiogenese/uso terapêutico, Ranibizumab/uso terapêutico, Panfotocoagulação, Acuidade visual

## Abstract

**Purpose:**

To compare visual acuity, macular thickness, and the area of active neovascularization based on fluorescein angiography outcomes associated with standard single-spot panretinal photocoagulation in the Early Treatment Diabetic Retinopathy Study (ETDRS) pattern combined with intravitreal ranibizumab injection versus multiple-spot full scatter (PASCAL) panretinal photocoagulation combined with intravitreal ranibizumab injection versus intravitreal injection alone in patients with proliferative diabetic retinopathy.

**Methods:**

Patients with proliferative diabetic retinopathy and no prior laser treatment were randomly assigned to receive three different types of treatment. Panretinal photocoagulation in the ETDRS group was administered in two sessions (weeks 0 and 2), and panretinal photocoagulation in the PASCAL group was administered in one session (week 0). Intravitreal injection of ranibizumab was administered at the end of the first laser session in both the ETDRS and PASCAL groups and at week 0 in the intravitreal injection group. Comprehensive ophthalmic evaluations were performed at baseline and every 4 weeks through week 48.

**Results:**

Thirty patients (n=40 eyes) completed the 48-week study period. After treatment, best-corrected visual acuity was significantly (p<0.05) improved at all follow-up visits in the group receiving intravitreal injection alone, at all but week 4 in the ETDRS group, and at all but weeks 4 and 8 for the PASCAL group. A significant decrease in central subfield macular thickness was observed in the PASCAL group at weeks 4, 8, and 48; only at week 48 in the intravitreal injection group; and never in the ETDRS group. There was no significant difference among the three treatment groups with respect to change from baseline to week 48 in best-corrected visual acuity, central subfield macular thickness, or fluorescein leakage from active neovascularization in best-corrected visual acuity, central subfield macular thickness, or fluorescein leakage from active neovascularization.

**Conclusions:**

Intravitreal injection alone or combined with single- or multiple- spot panretinal photocoagulation yielded similar outcomes with respect to mean change in best-corrected visual acuity, central subfield macular thickness, and fluorescein leakage from active neovascularization at up to one-year of follow-up. All subjects provided written informed consent to participate (NCT02005432 in clinicaltrials.gov).

## Abstract (pt)

**Objetivo:**

Comparar as medidas de acuidade visual, espessura macular central e área de neovasos ativos na angiofluoresceinografia submetidos a panfotocoagulação retiniana padrão ETDRS associado a injeção intravítrea de ranibizumabe versus panfotocoagulação padrão PASCAL associado a injeção intravítrea de ranibizumabe versus somente injeção intravítrea de ranibizumabe em pacientes com retinopatia diabética pro liferativa.

**Métodos:**

Pacientes com retinopatia diabética proliferativa e virgens de tratamento, randomicamente divididos nas três diferentes terapias retinianas. Panfotocoagulação no grupo ETDRS em 2 sessões (semanas 0 e 2) e no grupo PASCAL, na semana 0. Injeção intravítrea de ranibizumabe realizado ao fim da primeira sessão de laser em ambos os grupos: ETDRS e PASCAL, e na semana 0 no grupo injeção intravítrea de ranibizumabe. Avaliações oftalmológicas, tomografia de coerência óptica e angiofluoesceinografia realizados na visita basal e a cada 4 semanas por 48 semanas.

**Resultados:**

Trinta pacientes (n=40 olhos) completaram as 48 semanas de seguimento. Após o tratamento, a acuidade visual melhorou significantemente em todas a visitas no grupo injeção intravítrea de ranibizumabe (p<0,05); em todas exceto na semana 4 no grupo ETDRS, em todas exceto nas semanas 4 e 8 no grupo PASCAL. Redução significativa na espessura do subcampo central foi evidenciada no grupo PASCAL nas semanas 4, 8 e 48; somente na semana 48 no grupo injeção intravítrea de ranibizumabe, e em nenhuma visita no grupo ETDRS. Redução também na área de neovasos ativos em todas as visitas em todos os grupos. Não houve diferença significante entre os três grupos com relação a mudança media na medidas de acuidade visual, espessura macular central ou área de neovasos ativos da visita inicial para a semana 48.

**Conclusões:**

Somente IVB ou este associado a panfotocoagulação ETDRS ou PASCAL, apresentaram efeitos semelhantes em relação a medidas de acuidade visual, espessura do subcampo central e área de neovasos ativos no decorrer de 48 semanas de seguimento.

## INTRODUCTION

Diabetic retinopathy is the leading cause of visual loss and blindness among patients aged 20 to 70 years in the United States^(1)^. About 40% of patients older than 40 years diagnosed with diabetes mellitus show some form of diabetic retinal changes, with 8.2% exhibiting retinal damage that threatens vision, such as clinically relevant diabetic macular edema and advanced proliferative diabetic retinopathy (PDR)^(2)^.

The Diabetic Retinopathy Study and Early Treatment Diabetic Retinopathy Study (ETDRS) established panretinal laser photocoagulation (PRP) as the gold standard for the treatment of high-risk PDR, with treatment references such as a laser spot exposure time of 100-200 ms and the production of moderately white-grayish retinal spots with single laser shots in multiple sessions^(1^,^3)^. However, about 40% of patients with high-risk PDR do not respond to PRP^(4)^. In addition, PRP is associated with some adverse effects, including loss of the peripheral visual field, reduced dark adaptation, patient discomfort during the laser sessions, and, in some cases, macular edema with worsening of visual acuity^(5)^.

New laser treatment systems, such as the PASCAL (Pattern Scanning Laser, Topcon, Santa Clara, CA), have been designed to reduce laser-associated deleterious effects through the use of short laser pulses (i.e., 20 to 30 ms) and the application of multiple standardized spots in a single shot. Use of the pattern scanning laser for PRP has been reported to be associated with a reduction in the number of laser sessions and less patient discomfort^(6)^. The Manchester PASCAL group reported a reduced incidence of macular edema and less visual field loss over a 3-month follow-up period in patients treated for high-risk PDR with a single PASCAL session with a reduced pulse as compared with patients treated with a conventional laser pulse duration. With 18 months of follow-up, additional PASCAL sessions were required to achieve complete regression of retinal neovascularization^(7^,^8)^.

There have been recent investigations of PDR therapy that combines the long-lasting response typically associated with PRP with the more rapid-onset, although short-lasting, action of anti-vascular endothelial growth factor (anti-VEGF) agents^(9^-^11)^. In the IRaHi study, the use of PRP plus intravitreal ranibizumab (IVR) was associated with a greater reduction in the total area (mm^2^) of fluorescein leakage from active retinal neo vascularization at 48 weeks compared with the group treated with PRP only^(12)^. In the current study, three treatment techniques for patients with PDR are compared: standard single-spot PRP as described in the ETDRS combined with an intravitreal injection of 0.5 mg ranibizumab (the ETDRS-PRP+IVR group) versus multiple-spot full scatter (PASCAL) PRP combined with IVR (PASCAL-PRP+IVR group) versus IVR alone.

## METHODS

The study was approved by the Research Ethics Committee of Clinics Hospital, Faculty of Medicine of Ribeirão Preto, University of São Paulo (HCFMRP-USP), Brazil, and all subjects gave written informed consent to participate. Between March 2012 and November 2013, all adult patients with treatment-naive PDR and a best-corrected visual acuity (BCVA) better than 20/800 evaluated at the Retina and Vitreous Section of the Department of Ophthalmology, HCFMRP-USP, were invited to participate in the study.

### Exclusion criteria

Study exclusion criteria included the following: (1) presence of advanced PDR (i.e., vitreous hemorrhage that would prevent documentation of the funduscopic examination or administration of PRP) or presence of traction retinal detachment; (2) presence of ringshaped retinal neovascularization extending along both temporal arcades and the optic disc; (3) an abnormality of the vitreoretinal interface in the macular region that would lead the investigator to consider the necessity of pars plana vitrectomy; (4) intravitreal injection of corticosteroids or other antiangiogenic drugs during the prior 6 months; (5) inability of patient to fixate and perform reliable automated static perimetry; (6) history of cataract surgery within the previous 3 months; (7) history of pars plana vitrectomy or scleral buckle; (8) acute ocular infection; (9) allergy to fluorescein; (10) medical or psychological conditions that would prevent the patient from providing written informed consent or completing the study; (11) significant uncontrolled disease that, in the opinion of the investigator, would prevent the patient from completing the study; and (12) participation in another clinical study during the previous 30 days.

During the recruitment phase, 50 consecutive patients who met the aforementioned inclusion and exclusion criteria were enrolled into the study. At the baseline visit, each patient underwent detailed ophthalmologic assessment, including BCVA measurement according to standardized ETDRS refraction protocols using modified ETDRS cards 1, 2, and R, as well as applanation tonometry, slit-lamp biomicroscopy examination under mydriasis (including classification of crystalline opacity status using the Lens Opacities Classification System [LOCS III])^(13)^, and indirect funduscopic examination.

Digital ocular stereoscopic fundus photographs (TRC50DX; IMAGEnet, Topcon, Tokyo, Japan), fluorescein angiography, and optical coherence tomography (OCT; HRA-OCT, Heidelberg, Germany) images were obtained.

### Randomization and treatment groups

Patients were randomly assigned, based on a computer-generated sequence, to one of the following three treatment groups.

*ETDRS-PRP+IVR group:* patients assigned to this group were treated with single-spot full-scatter PRP with a PUREPOINT green diode laser (Alcon, Fort Worth, TX) in combination with an intravitreal injection of 0.05 mL (0.5 mg) ranibizumab (Lucentis^®^) 180 minutes after the first laser session (week 0). In this group, laser treatment was performed in two sessions (at week 0 and week 2) and consisted of 800 to 900 shots, for a total of 1600 to 1800 shots with a pulse duration of 100 ms and power modulated to generate moderately white spots on the retina^(2)^.

*PASCAL-PRP+IVR group:* Patients assigned to this group were treated with multiple-spot full-scatter PRP with a PASCAL standardized scan laser (532 µm; OptiMedica, Santa Clara, CA) in combination with an intravitreal injection of 0.05 mL (0.5 mg) ranibizumab (Lucentis^®^) 180 minutes after the first laser session (week 0). In this group, laser treatment was performed in a single session (week 0) consisting of 1300 to 1800 spots, with a pulse duration of 20 ms, 5 × 5 multispot array, and 1.5 burn width to generate moderately white spots on the retina^(14)^.

In the above groups, PRP was performed using an Ocular Mainster PRP 165 lens with a dynamic field of view of 180° and using a 200-µm spot size (which produces a 392-µm spot size on the retina). IVR injections were performed 180 minutes after the first laser session (week 0) by a single retina specialist.

*IVR group:* Patients assigned to this group were treated with an intravitreal injection of 0.05 mL (0.5 mg) ranizumab (Lucentis^®^) at week 0.

### Intravitreal injection

Intravitreal injections were performed in a clinic setting 180 minutes after PRP (except for the IVR group) using a disposable syringe with a BD Ultra-Fine™ 29G ½-inch needle, via the pars plana 3.5 mm posterior to the limbus, using topical anesthesia. After the procedure, optic nerve perfusion was assessed by indirect binocular ophthalmoscopy, with paracentesis of the anterior chamber considered in cases of poor perfusion. After injection, patients were instructed to use antibiotic eyedrops (0.5% moxifloxacin), in accordance with the drug label (one drop every 4 hours for 1 week), in the eye that received the intravitreal injection.

### Diabetic macular edema management

At week 0, in eyes with clinically significant diabetic macular edema (CSME), a laser was applied in a grid pattern (20-ms duration laser pulses with a Volk PDT laser disk, spot size of 112.5 µm [75 × 1.5 lens magnification], and sufficient power to cause weakly visible marks with a PUREPOINT green laser diode [Alcon]). Eyes with CSME were considered those with at least one of the following: (1) retinal thickening within 500 µm from the center of the macula, (2) hard exudates within 500 µm from the center of the macula associated with thickening of the adjacent retina, and (3) one or more zones of retinal thickening with an area of 1 disc diameter (1.5 µm), with at least one part located inside 1 disc diameter from the center of the macula.

### Ophthalmologic evaluations

**Figure 1:**
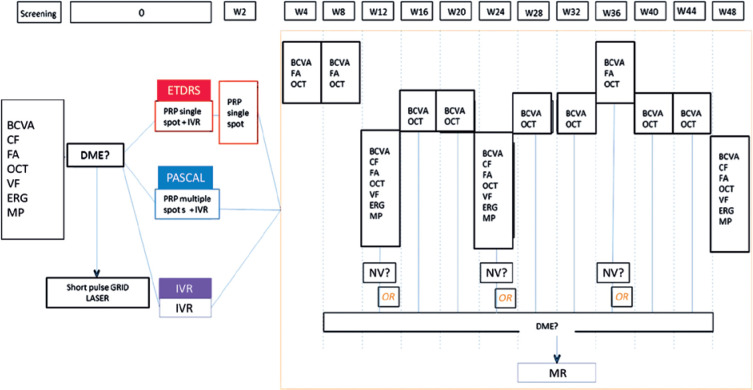
Flow diagram of the examinations conducted in each treatment group over 1 year of follow-up.

Comprehensive ophthalmic evaluations, including ETDRS BCVA and central subfield thickness (CSFT) as measured by spectral domain OCT, were performed at baseline and every 4 weeks through week 48. The area of fluorescein leakage from active new vessels (FLA) was measured by fluorescein angiography at baseline and at weeks 4, 8, 12, 24, 36, and 48. Fluorescein angiography pictures were taken 2.5 to 3.0 minutes after the injection of fluorescein dye. Local and systemic adverse effects, including changes in intraocular pressure and crystalline status, were monitored throughout the study (Figure 1).

### Retreatment criteria

At follow-up visits at week 12 through week 44, patients were treated with an injection of IVR (0.5 mg in 0.05 mL) if fluorescein angiography demonstrated the presence of actively leaking retinal neovascularization and/or if OCT demonstrated a CSFT of greater than 300 µm.

### The sample size

The sample size was estimated based on the variation in FLA leakage (main outcome) after combined LASER and anti-VEGF injection treatment for high-risk PDR patients from a previous study^(12)^. Considering a standard deviation of 1.7 mm^2^, a sample of 45 eyes (15 per group) would be necessary for an 80% chance to detect a 2-mm^2^ difference between groups.

### Statistical analysis

Baseline data were compared using one-way analysis of variance followed by Tukey-Kramer testing for multiple mean comparisons, whereas group comparisons during follow-up were performed using analysis of covariance with “group”, “time”, and “group × time” as effects, followed by Tukey honestly significant difference testing. Calculations were performed using JMP 10.0 (SAS). The significance level was set at *p*<0.05.

## RESULTS

**Figure 2:**
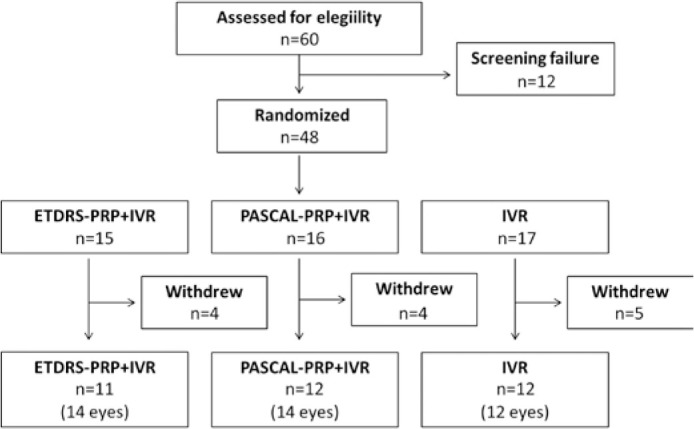
Flow diagram of patient follow-up.

Thirty of the 50 patients enrolled in the study (40 eyes) completed the 48 weeks of follow-up (Figure 2; Table 1). Demographic characteristics of the study participants are summarized in table 2. There was no significant difference among the groups with respect to age, duration of diabetes mellitus, level of glycosylated hemoglobin, area of active retinal neovascularization, BCVA, or CSFT.

**Table 1:** Reasons for patient loss to follow-up.

Number of excluded patients	Reason	Group	Week visit
7	Missing 2 consecutive visits	-	-
1	Died of acute myocardial infarction	PASCAL	24
1	Underwent surgery for cardiac revascularization	ETDRS	40
1	Underwent vitrectomy due to persistent vitreous hemorrhage in the left eye	PASCAL	20
1	Vitrectomy for a nasal combined rhegmatogenous/tractional retinal detachment	PASCAL	44
2	Worsening renal function	IVR; IVR	2; 24
2	Diabetic foot surgery	ETDRS; ETDRS	8; 12
1	Arm fracture	IVR	32
1	Sepsis due to urinary infection	IVR	8
3	Missing two or more scheduled IVR injections	ETDRS; PASCAL; IVR	16; 24; 44

**Table 2:** Baseline data.

Groups (n)	ETDRS + IVR (14)	PASCAL + IVR (14)	IVR (12)	p value
Age	57.0 ± 3.4	58.5 ± 3.1	50.6 ± 3.3	0.2042
Duration of DM (years)	16.1 ± 2.5	11.3 ± 2.6	9.8 ± 2.4	0.1808
HbA1c (%)	10.2 ± 1.5	11.0 ± 1.3	9.0 ± 1.4	0.5848
FLA (mm^2^)	21.0 ± 5.6	15.5 ± 5.9	17.1 ± 5.5	0.7790
BCVA (logMAR)	0.500 ± 0.086	0.492 ± 0.093	0.464 ± 0.086	0.9547
CSFT (µm)	283.4 ± 24.1	318.0 ± 26.0	369 ± 24.1	0.0523

IVR= intravitreal ranibizumab; HbA1c= glycosylated hemoglobin (hemoglobin A1c); PRP= panretinal photocoagulation; FLA= fluorescein leakage area; BCVA= best-corrected visual acuity; CSFT= central subfield thickness.

Twelve of 40 eyes (3/14 in the ETDRS group, 7/14 in the PASCAL group, 2/12 in the IVR group) were submitted to grid macular laser for CSME at the baseline visit. After laser treatment, patients were assigned to their respective treatment groups. At the week 48 visit, 3 of 40 eyes showed a CSFT >300 µm (1/14 in the ETDRS group, 2/14 in the PASCAL group, 0/12 in the IVR group).

During the 48 weeks of the study, no serious adverse events related to IVR were detected in any patient, with the procedure being well tolerated and no clinical evidence of uveitis, endophthalmitis, or ocular toxicity. Furthermore, there were no significant changes observed in crystalline lens status or intraocular pressure. Minor localized events related to IVR, such as subconjunctival hemorrhage, were reported in 5% of the patients.

### Fluorescein leakage area of active new vessels (FLA)

**Figure 3:**
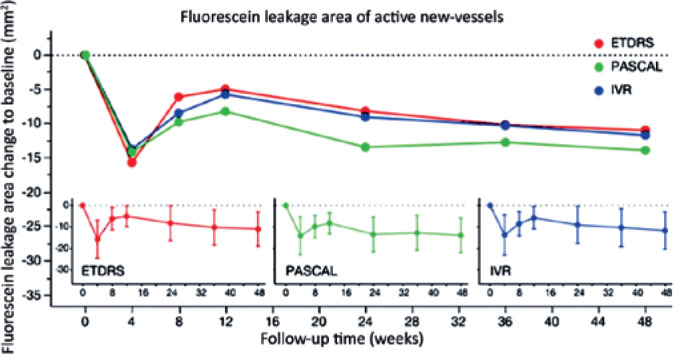
Circles represent the mean change in fluorescein leakage area (FLA) compared with baseline at each follow-up visit. Red, green, and blue represent the means from the ETDRS-PRP, PASCAL-PRP, and IVR groups, respectively. The inset graphs at the bottom show the mean ± 95% confidence limit at each follow-up visit for each group.

No significant difference in FLA was observed among the ETDRS-PRP+IVR, PASCAL-PRP+IVR, and IVR groups at baseline (Table 2). The mean ± standard error (SE) FLA (mm^2^) was 21.0 ± 5.6, 17.1 ± 5.5, and 15.5 ± 5.9, respectively (Figure 3). Intragroup analysis revealed a significant FLA reduction at all follow-up visits compared with baseline. At week 48, there was no significant difference among the treatment groups in FLA reduction (*p*=0.8519).

### BCVA

**Figure 4:**
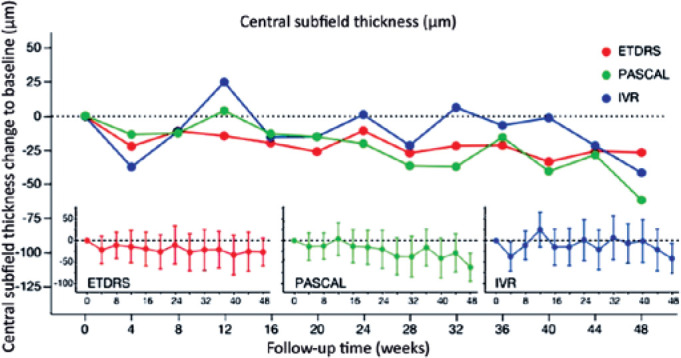
Circles represent the mean change in CSFT compared to baseline at each follow-up visit. Red, green, and blue represent the means from the ETDRS-PRP, PASCAL-PRP, and IVR groups, respectively. The inset graphs on the bottom show the mean ± 95% confidence limit at each follow-up visit for each group.

No significant difference in baseline BCVA was observed among the ETDRS-PRP+IVR, PASCALPRP+IVR, and IVR groups. Mean ± SE BCVA (logMAR) was 0.5 ± 0.086, 0.464 ± 0.086, and 0.492 ± 0.093, respectively (Table 2). BCVA was significantly improved from baseline at all follow-up visits in the IVR group; at weeks 8, 12, 16, 20, 24, 28, 32, 36, 40, 44, and 48 in the ETDRS-PRP group; and at weeks 16, 20, 24, 28, 32, 40, 44, and 48 in the PASCAL-PRP group. No significant difference in BCVA improvement was observed among the treatment groups at 48 weeks (*p*=0.4185; Figure 4).

### CSFT

**Figure 5:**
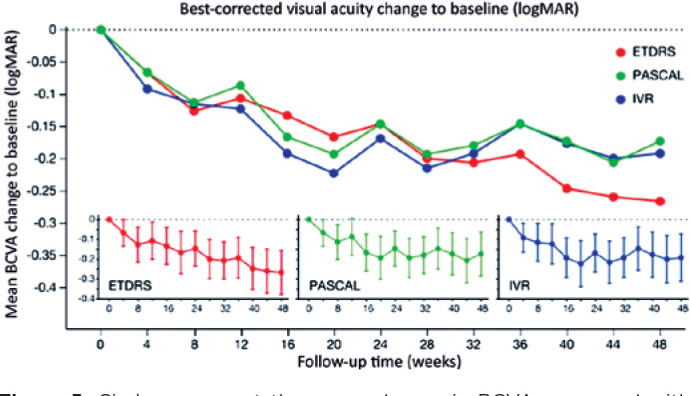
Circles represent the mean change in BCVA compared with baseline at each follow-up visit. Red, green, and blue represent the means from the ETDRS-PRP, PASCAL-PRP, and IVR groups, respectively. The inset graphs on the bottom show the mean ± 95% confidence limit at each follow-up visit for each group.

No significant difference in baseline CSFT was observed among the ETDRS-PRP+IVR, PASCALPRP+IVR, and IVR groups. Mean ± SE CSFT (µm) was 283.4 ± 24.1, 369 ± 24.1, and 318.0 ± 26, respectively (Table 2). There was a significant reduction of CSFT at weeks 4, 8, and 48 in the PASCAL-PRP+IVR group and only at week 48 in the IVR group, with no significant reduction in the PASCAL-PRP+IVR group. No significant difference in CSFT reduction was observed among the three treatment groups at week 48 (p*=*0.1251; Figure 5).

### Number of IVR injections during the 48 weeks of the study

**Figure 6:**
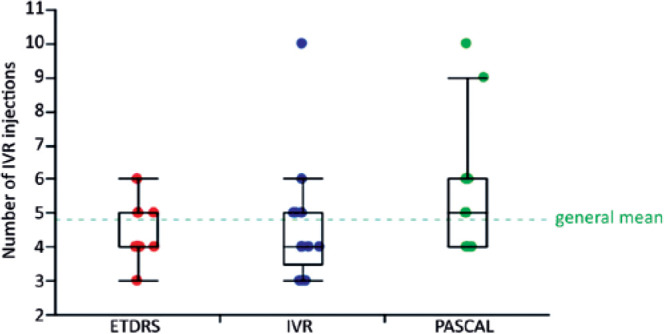
Circles represent the mean number of IVR injections performed in each patient. Red, green, and blue represent the means from the ETDRS-PRP, PASCAL-PRP, and IVR groups, respectively. The green dashed line is the general mean, the line within the box represents the median, and the ends of the box are the 75th and 25th quantiles. The whiskers extend to the upper and lower data point values (not including outliers: outside 1.5 × interquartile range).

There was no significant difference among the treatment groups with respect to the number of IVR injections administered during the study period. The median number of IVR injections in patients without CSME was four for all three treatment groups, and in patients with CSME, the median number of IVR injections was 5, 6, and 5 for the ETDRS-PRP+IVR, PASCALPRP+IVR, and IVR groups, respectively (Figure 6).

## DISCUSSION

In the current study, after 1 year of follow-up, the three different therapeutic strategies were associated with a significant reduction in FLA in patients with PDR. A previous study by our group^(10)^ also showed a significant reduction in the area of persistent retinal new vessels at 1, 6, 12, 24, 36, and 48 weeks after IVR, with IVR being administered every 4 months if fluorescein leakage due to active new vessels was detected by fluorescein angiography. In another study by our group^(12)^, comparing ETDRS PRP to ETDRS PRP in combination with IVR for high-risk PDR, a reduction in active new vessel area was observed by fluorescein angiography in both treatment groups; the reduction, however, was greater in the group that received combination therapy (-2.9 mm^2^ ± 1.3 in the PRP group and -5.8 mm^2^ ± 0.7 in the PRP plus IVR group; p=0.0291).

According to Vander et al.^(4)^, PRP was associated with complete regression of retinal neovascularization at 12 weeks of follow-up in 60% of patients (39 eyes). In a more recent European study reported by Figueira et al.^(15)^, eyes with high-risk PDR were randomly assigned to treatment with either PRP, IVR, or combination therapy of PRP+IVR, and the primary outcome was the proportion of eyes in which complete regression of neovascularization was achieved at 12 months. Complete regression of neovascularization elsewhere was achieved at 12 months in 30.8% (4/13), 37.5% (3/8), and 44.4% (4/9) of eyes in the PRP, IVR, and PRP+IVR groups, respectively. Complete regression of neovascularization of the disc was achieved at 12 months in 22.2%, 40.0%, and 37.5% of eyes in the PRP, IVR, and PRP+IVR groups, respectively. In the current study, as well as in a previous study by our group^(12)^, no patient achieved complete regression of retinal neovascularization at week 48 despite combined LASER plus anti-VEGF therapy. We believe that the higher rate of persistent neovascularization in these two studies compared with the aforementioned European study^(15)^ may be due, at least in part, to the poor glycemic control of the Brazilian study participants (the mean baseline hemoglobin A1c of participants in the current study was 10.2%, 11.0%, and 9.0% in the ETDRS+IVR, PASCAL+IVR, and IVR groups, respectively). Indeed, Boltri et al.^(16)^ reported worse glycemic control in Hispanic patients compared with white non-Hispanic patients.

In the current study, there was a significant improvement in mean BCVA from baseline to week 48 in all three treatment groups. Ramos-Filho et al.^(12)^ reported that the BCVA improvement in patients treated with combined PRP+IVR after a 48-week follow-up period was significantly greater than that of patients who received only PRP (5.8 logMAR units for the PRP+IVR group versus 2.9 logMAR units for the PRP group; p=0.0291). Similarly, the Diabetic Retinopathy Clinical Research Network reported that, in Protocol S (which compared PRP versus IVR for treatment of PDR)^(17)^, the improvement in visual acuity at 2 years was significantly greater in the IVR group compared with the PRP group (mean change in visual acuity letter score of +4.5 in the IVR group versus -0.3 in the PRP group, p<0.001). In the 5-year report of the same study^(18)^, the difference in BCVA improvement was no longer verified (the mean [SD] change in visual acuity letter score was 3.1 [14.3] and 3.0 [10.5] letters, respectively; adjusted difference, 0.6; 95% confidence interval, -2.3 to 3.5; p=0.68). However, for the 5-year follow-up analysis, only 66% of enrolled patients were included. Consequently, the study power and the strength of the conclusions decreased, and caution should be taken while considering these results. Moreover, taking into account the time of follow-up, the 2-year results from Protocol S are more feasible for comparison with the one-year BCVA results from the present study. In the study by Figueira et al.^(15)^, there was no significant change in visual acuity in any of the study groups during 12 months of follow-up.

The current study did not detect a significant difference in CSFT reduction among the treatment groups (-22.33 ± 16.03 for the ETDRS-PRP+IVR group, -62.27 ± 16.03 for the PASCAL-PRP+IVR group, and -41.77 ± 17.21 for the IVR group; p>0.05). Although the absolute value of the CSFT reduction was greater in the PASCAL-PRP+IVR group, this is likely due, at least in part, to the higher baseline CSFT in this group. Other studies suggest that the use of IVR alone or in combination with PRP leads to better results regarding CSFT reduction when compared with PRP alone. In the study by Ramos-Filho et al.^(12)^, patients with PDR treated with PRP plus IVR maintained a stable CSFT after 48 weeks of follow-up (mean change: -14.7 ± 39.1 µm; p=0.0698), whereas patients treated with PRP only demonstrated a significant increase in CSFT after the same follow-up period (mean change: 18.1 ± 9.4 µm; p=0.0043). In the study by Figueira et al.^(15)^, CSFT remained stable in all study groups at all follow-up visits. In DRCR Protocol S^(17)^, the mean change in CSFT from baseline to 2 years was -47 µm (±SD) in the IVR group and -3 µm (±SD) in the PRP group (p<0.001). In summary, the results of these studies are consistent with the hypothesis that the reduction in macular thickness is more pronounced in groups treated with combined PRP and IVR or IVR alone than in groups treated with PRP alone. More recently, the 5-year report of Protocol S^(18)^ did not show a difference in CSFT reduction among PDR patients treated with PRP versus IVR. However, as mentioned above, the sample size reduction for this long-term analysis limits its strength and reliability. Also, the 2-year CSFT values from Protocol S are more feasible than the 5-year values for comparison with the one-year CSFT results of the present study.

In patients with CSME and in patients without CSME, there was no significant difference among the treatment groups with respect to the number of IVR injections needed to treat macular edema during the study period. In the two PRP groups, there was no worsening of edema that required an increased number of injections. This suggests that, in contrast to treatment with PRP alone, combination PRP+IVR therapy prevents development or exacerbation of macular edema. This is consistent with the study by Ramos-Filho et al.^(12)^, who reported that, after a 48-week follow-up period, patients with PDR treated with PRP+IVR maintained a stable CSFT, whereas patients treated with PRP alone demonstrated a significant increase in CSFT. Perhaps the addition of an anti-VEGF agent at the time of the laser-induced inflammatory insult prevents the macular edemainducing effects of released cytokines^(19^,^20)^, such that patients treated with PRP+anti-VEGF become similar to patients treated with anti-VEGF alone with respect to the number of intravitreal injections necessary to control macular edema^(21)^.

There was also no difference among the treatment groups with respect to the number of IVR injections needed to control retinal neovascularization. Panretinal photocoagulation and consequent destruction of the outer retina did not reduce the number of IVR injections needed to control neovascularization. In fact, in other studies reported by our group^(9^,^10^,^12)^, the use of anti-VEGF alone for treatment of persistent new vessels or combined PRP+anti-VEGF therapy for high-risk PDR patients, did not result in control of retinal neovascularization at 1 year in study populations with generally poor glycemic control. It is possible that a follow-up period of 1 year is too short to detect a reduced number of anti-VEGF injections needed to control neovascularization; studies of longer duration might detect the potential advantage of the theoretically permanent reduction in VEGF production^(22)^ induced by PRP in patients treated with combined PRP+anti-VEGF. Similarly, it is unknown whether a longer follow-up duration may reveal a potential benefit of a reduced number of anti-VEGF injections needed to control neovascularization when more intense laser therapy is performed, such as in the ETDRS-PRP group, compared with a less intense laser therapy, such as the PASCAL-PRP group.

The present study has some limitations. It was powered to detect a difference in FLA among groups, and conclusions about all other outcomes may be limited by the small number of patients included. A higher number of patients were excluded from the study than expected, and we ended up with a sample size of 40 patients instead of 45. For this reason, the difference in FLA among groups that could be detected by this study was increased from 2 mm^2^ to 2.3 mm^2^.

In summary, in the current study, when using IVR for the treatment of patients with PDR, the addition of ETDRS-PRP or PASCAL-PRP did not alter the control of retinal new vessels after 1 year of follow-up.
